# National estimates of the impact of electronic health records on the workload of primary care physicians

**DOI:** 10.1186/s12913-016-1422-6

**Published:** 2016-05-10

**Authors:** Jaeyong Bae, William E. Encinosa

**Affiliations:** School of Nursing and Health Studies, Northern Illinois University, Wirtz Hall 257, Dekalb, IL 60115 USA; Center for Delivery, Organization and Markets, Agency for Healthcare Research and Quality, 5600 Fishers Ln, Rockville, MD 20857 USA; McCourt School of Public Policy, Georgetown University, Washington, DC USA

**Keywords:** Electronic health records, Physician workload, Efficiency of care, Primary care

## Abstract

**Background:**

Eighty-four thousand primary care physicians have received $1.3 billion in HITECH payments for EHR adoption. However, little is known about how this will impact primary care workload efficiency and the national primary care shortage. This study examines whether EHR is associated with increases in face time with the patient per visit and increases in the physician’s patient volume per week.

**Methods:**

We used a nationally representative sample of 37,962 patient visits to 1470 primary care physicians during the pre-HITECH years 2006–2009 from the restricted-access version of the National Ambulatory Medical Care Survey. Quantile regressions were used to estimate the effects of EHR use on patient face time per visit and physician’s patient volume per week at different points of the time and volume distributions.

**Results:**

Primary care physicians with EHR spend an extra 1.3 face time minutes per visit, or 1.5 extra hours per week. This is 34,000 extra hours of face time per week in the U.S. However, physician age matters. Among young physicians, EHR use is associated with a decline in weekly patient volume, while EHR use among older physicians is associated with an increase in volume, regardless of initial practice size. If younger physicians behaved like older physicians when adopting EHR, there would be 37,600 additional patient visits per week in the U.S., the equivalent of adding 500 more primary care physicians to the U.S. workforce.

**Conclusion:**

EHR can enhance productivity/efficiency in primary care physician workloads.

## Background

Primary care physicians are playing essential care coordination roles in a number of recent health care reforms such as patient-centered care [1, 2], accountable care organizations (ACOs) [3], and medical homes [2]. However, the ongoing shortage of primary care doctors in the U.S. is a major challenge to the success of these reforms. The number of office visits to primary care physicians is projected to increase from 462 million in 2008 to 565 million in 2025 [4]. A recent Senate report indicates that 16,000 additional primary care physicians are required to meet the current need, and the shortage is predicted to increase to 52,000 physicians by 2025, mainly due to the coverage expansion through Medicaid and the Federal and State Marketplace exchanges under the Patient Protection and Affordable Care Act (ACA) [5].

Moreover, as the number of clinical guidelines increase for primary care in the shift away from specialty care, more time demands will be placed on primary care physicians. Yarnall et al. (2009) estimate that in order to implement all current national clinical guidelines for acute care, preventive care, and chronic care, primary care physicians would need to work 21.7 hours per day [6]. Primary care physicians have expressed this concern---38 % report not spending enough time with their patients during a typical office visit (Center for Studying Health System Change, 2008) [7]. This has not gone unnoticed by the patient. Only 75 % of patients thought that doctors always spend enough time with them during the office visit (AHRQ, 2012) [8]. Other studies also found that insufficient physician’s time with patients is associated with lower patient satisfaction and quality of care [9–11].

Health Information Technology (HIT) such as Electronic Health Records (EHRs) has the potential to address the primary care workforce shortage by improving the efficiency of primary care practices and the productivity of primary care physicians. In particular, when used effectively, EHRs increase the efficiency of healthcare delivery in primary care through enhancing workflow and decreasing redundant or inappropriate care [12–14]. To facilitate potential benefits due to the implementation and appropriate use of EHRs, the 2009 Health Information Technology for Economic and Clinical Health (HITECH) Act established the Medicare and Medicaid EHR program to encourage physicians to adopt the meaningful use of EHRs. As of June, 2013, 84,000 primary care physicians had received $1.3 billion in Medicare incentive payments for meaningful use EHR adoption [15]. Consequently, the basic EHR adoption rate for family physicians increased from 24.8 to 66.4 % during the period of 2005 to 2011 [16].

There is little research on how this HITECH expansion of EHR among primary care will improve primary care workload efficiency. While a few studies have found mixed results on the impact of EHR on patient face time with the physician, no research has yet examined the complete workload picture: time spent per visits and overall number of visits per week per physician [14, 17, 18]. It has been estimated that each office visit requires an additional 7 min of administrative work by the physician outside of the visit, amounting to about 7.8 extra hours a week [19, 20]. Thus, if EHR could reduce this administrative time, more patients could be seen per week and more time could be spent per visit. In this paper, we use nationally representative data to examine the effect of EHR use on overall primary care physician workloads in terms of patient visits per week and time spent per visit. We also examine how these effects vary across younger and older physicians.

## Methods

### Data source

We used the 2006–2009 National Ambulatory Medical Care Survey (NAMCS). The NAMCS is a cross-sectional national probability sample survey administrated by the National Center for Health Statistics (NCHS) for the Centers for Disease Control and Prevention (CDC) [21]. The NAMCS collects data on patient office visits to non-federally employed office-based physicians in the United States. For each visit, physicians or staff members complete a one page survey form containing patient demographics, reasons for the visit, physicians’ diagnoses, and physicians’ time spent in face-to-face patient care. Detailed descriptions of the survey are available from the authors and on the NAMCS website (http://www.cdc.gov/nchs/ahcd.htm). We examined 37,962 patient visits to 1470 primary care physicians during 2006–2009 (general practitioners, family practitioners, and general internists). Through the NCHS Research Data Center, we have obtained access to a restricted version of the NAMCS data with information on physician’s age and the number of patient visits per physician per week. We use 2006–2009 since it is the baseline period prior to the introduction of the HITECH EHR adoption subsidies.

### Patient face time and patient volume

To gauge productivity/efficiency gains in primary care physician workloads, we use (1) duration of time measured in minutes of the face-to-face encounter between physicians and patients (patient face time) for direct patient care during the office visit, and (2) number of total patient office visits per physician per week (patient volume). Patient face time, self-reported by physicians, only includes time physicians spent with their patients. Time spent waiting to see physicians, reviewing patients’ medical records by physicians before seeing patients, and receiving care from non-physician clinicians are excluded. Physician’s number of patient visits per week is the count of patient visits during the survey week reported by physicians or office staffs.

### Basic EHR use

The key independent variable of this study is a binary EHR adoption variable “basic EHRs” defined for NAMCS data by DesRoches et al. [22] The basic EHRs include at least 5 fundamental EHR functions: (1) patient demographic information, (2) clinical notes, (3) computerized orders for prescriptions, (4) viewing laboratory results, and (5) viewing image results.

### Statistical methods

A cross-sectional analysis of pooled survey data was conducted. Multivariate regression techniques were used to estimate the impact of the basic EHR use on the following outcomes of interest: patient face time per visit and physician’s number of patient visits per week. Ordinary least square (OLS) regressions will only capture the effects of EHR at the mean visit time and mean patient volume. But, the effect of EHR on face time may vary depending on whether the visit is particularly short or long, and the effect on patient volume may vary depending on whether the physician has a small or large practice. To capture this variation in the effects of EHR, a novel feature of this paper is that we use simultaneous-quantile regressions (SQR) in Stata 12 [23]. In our visit-level face time regressions, SQR simultaneously estimates the impact of EHR on face time at five points in the distribution of face time: 10 min (12^th^ percentile), 15 min (15^th^ percentile), 20 min (60^th^ percentile), 25 min (75^th^ percentile), and 30 min (80^th^ percentile). Standard errors in SQR are bootstrapped. In the physician-level patient volume regressions, SQR simultaneously estimates the impact of EHR on volume at three points in the distribution of patient volume: the 10^th^, 50^th^, and 90^th^ quartiles of patient volume (20, 64, 120 visits per week). Time and volume are logged in all regressions.

Next, in each regression we construct indicators for young physicians (age 36 or younger) (12^th^ percentile) and older physicians (age 62 or older) (88^th^ percentile) and include interaction terms between these two indicators and ‘basic EHR’ to examine how the effect of the basic EHR use on physicians’ workload productivity varies across physician’s age. In addition to these interactions, our regressions control for 46 covariates: patient characteristics, health status of patients, visit characteristics, physicians characteristics, physician group characteristics, and other covariates such as geographic region, metropolitan statistical area (MSA) status, and survey year. MSA is delineated by the US Office of Management and Budget Census as a “core urban area of 50,000 or more population." MSA has been commonly used in literature to define urban/rural status. All the covariates with descriptive statistics are reported in Appendix 1.

## Results

### Raw trends in basic EHR use, patient face time, and patient volume by physician age

During 2006–2009, 19.9 % of primary care physicians had basic EHRs. Older physicians were less likely to adopt basic EHRs than younger physicians (column 1, Table 1). While 23.1 % of physicians aged 36 or less and 20.4 % of physicians aged between 37 and 61 had basic EHRs, only 13.6 % of physicians aged 62 or older had basic EHRs. Physicians with basic EHRs spent longer face time with their patients than physicians without basic EHRs across all physician age groups (column 2, Table 1). In general, under EHR adoption, physicians had fewer patient visits per week than without EHR. However, this was reversed for older physicians, who had more patient visits with EHR than without (column 3, Table 1). Moreover, older physicians that adopted EHR had more visits per week than the young and middle-aged physicians that adopted. In the case of non-EHR adopting physicians, older physicians had fewer visits per week than the young and middle-aged physicians. Thus, the raw data indicates that EHRs are associated with increases in the physician’s face time with the patient regardless of physician age, whereas the association between EHRs and patient volume varies across physician age. Next, we see if these patterns persist once we control for patient and physician characteristics in multivariate regressions.

**Table 1:** Basic EHR use, patient face time, and physician’s number of patient visits per week by physician age (Unadjusted)

	Basic EHR use^a^	Patient face time per visit, in minutes^b^, Mean (SD)	Physician’s number of patient visits per week^a^, Mean (SD)
All physicians	Yes: 19.9 % No: 80.1 %	20.4 (12.2) 19.0 (11.1)^c^	67.5 (35.7) 69.7 (46.4)^c^
Younger physicians (12 %)	Yes: 23.1 % No: 76.9 %	20.1 (10.0) 18.8 (11.6)	62.3 (30.0) 65.1 (36.1)^c^
Middle-aged physicians (76 %)	Yes: 20.4 % No: 79.6 %	20.5 (12.4) 18.9 (11.1)^c^	67.8 (36.0) 72.3 (48.3)^c^
Older physicians (12 %)	Yes: 13.6 % No: 86.4 %	20.9 (13.5) 19.4 (10.5)	73.7 (41.2) 58.6 (42.3)^c^

Note: Middle age is 37 to 61

^a^Basic EHR use and physician’s patient visits per week are at the physician level

^b^Patient face time in minutes is at the visit level

^c^The “Yes” category is significantly different from the “No” category at the *p* < .05 level

### Estimated association between EHR use and patient face time

**Fig. 1:**
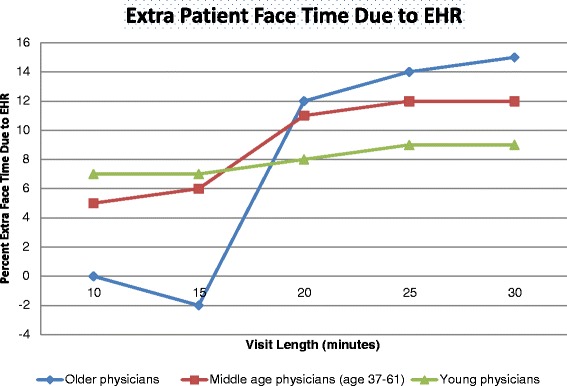
The estimated effect of EHR on patient face time. Note: Effects were estimated using the Table 2 quantile regressions controlling for patient and physician characteristics. All points are significantly different from zero at the *p* < .05 level except for 10 and 15 min visits for older physicians

Figure 1 presents the impact of basic EHR on patient face time at selected levels of patient face time (10, 15, 20, 25, 30 min) by physician age group, estimated by quantile regressions controlling for 46 covariates (the regressions are detailed in Appendix 2). Figure 1 is presenting the full incremental effects of EHR estimated from Table 2.^1^ In Fig. 1, young physicians (age 36 or younger) using basic EHR consistently increased patient face time by 7-9 % at all points in the time distribution (*p* < .01). On the contrary, older physicians did not increase patient face time for visits of 10 min and 15 min (statistically insignificant), but raised patient face time by 12 %, 14 %, and 15 % for patient visits of 20 min, 25 min, and 30 min, respectively (*p* < .01).

**Table 2:** Quantile regression estimates of the impact of EHR on patient face time per visit

Variables	At mean visit	10 minute visit	15 minute visit	20 minute visit	25 minute Visit	30 minute visit
Key independent variables						
Basic EHR	0.062*** (0.007)	0.053*** (0.012)	0.054*** (0.009)	0.104*** (0.012)	0.112*** (0.011)	0.117*** (0.011)
Young physician	-0.009 (0.008)	0.009 (0.010)	0.010 (0.009)	-0.006 (0.009)	-0.032*** (0.010)	-0.033*** (0.010)
Older physician	0.007 (0.008)	-0.014 (0.012)	-0.001 (0.012)	0.049*** (0.012)	0.026** (0.013)	0.030* (0.018)
Basic EHR * Young physician	0.005 (0.016)	0.016 (0.025)	0.009 (0.021)	-0.027* (0.015)	-0.030 (0.023)	-0.034 (0.024)
Basic EHR * Older physician	0.006 (0.020)	-0.053** (0.021)	-0.073*** (0.026)	0.006 (0.031)	0.020 (0.051)	0.025 (0.048)
*N* = 37,962						

Note: Visit-level data. Standard errors are in parentheses. Coefficients on other covariates are available in Appendix 2. Minutes are logged

***p value < .01

**p value < .05

*p value < .1

Overall, in terms of absolute magnitude, these effects can be expressed as follows: for 15 min visits, older physicians spent 1.4 min less per visit than younger physicians due to EHR, but for 30 min visits, they spent 1.8 min more than younger physicians due to EHR. Thus, compared to younger physicians, there is a 3.2 min increase in face time over the range of visits (15 to 30 min) for older physicians that is entirely due to EHR. Note that at the mean visit time (19 min), young and old physicians behave about the same, spending 7 % more time with the patient (1.3 min, *p* < 0.001) under EHR than without EHR.^2^ These extra 1.3 face time minutes per visit result in a total of 1.5 extra hours of face time per week under EHR. In the subsequent section, we will examine whether the increase in patient face time causes physicians to reduce the number of visits over the week.

### Estimated association between EHR use and the physician’s number of patient visits per week

**Fig. 2:**
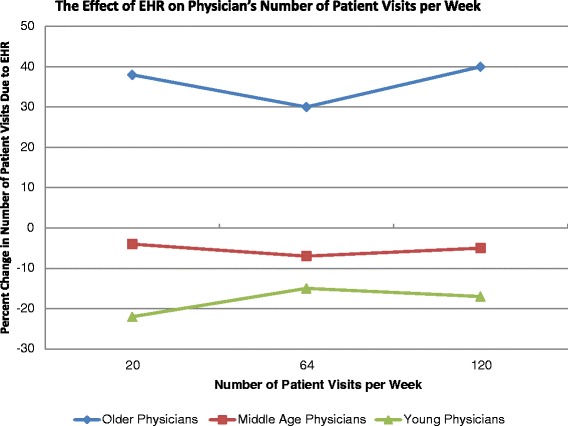
The effect of EHR on physician’s number of patient visits per week. Note: Effects were estimated using the Table 3 quantile regressions controlling for patient and physician characteristics. The average effect for older physicians is significantly different from zero at *p* = 0.02. The average effect for young physicians is significantly different from zero at *p* = 0.09. The average effect for middle age physicians is not significantly different from zero

Figure 2 shows the effect of basic EHR use on the physician’s number of patient visits per week at the 10^th^, 50^th^, and 90^th^ quantiles of patient volume (20, 64, 120 visits per week) by physician age group, estimated by quantile regressions (see Table 3). While basic EHR use reduced the young physician’s number of patient visits by 17-22 %, older physicians increased number of visits by 30-40 %, consistently across all scales of practice sizes. This indicates that EHRs are associated with increased weekly patient volume among older physicians and with reduced patient volume among young physicians. In column 1, Table 3, the overall average impact of EHR on the young is the sum of the coefficients: EHR + EHR*young = -.027-.185 = -.212. Not shown, the standard error for this sum is estimated to be .126, so the full effect of -0.212 has a *p*-value < 0.09. Log retransformed, this is an effect of size -.19 as the average effect of EHR on the number of weekly visits for young physicians. For older physicians, the average effect is + .44 (*p* = 0.015). This amounts to an increase of 26 visits per week for older physicians with EHR compared to older physicians without EHR. For younger physicians, EHR is associated with a reduction of about 12 visits per week at the mean. Overall, young physicians with EHR have 53 visits per week compared to 85 visits for older physicians with EHR. When there is no EHR adoption, young physicians have 65 visits per week compared to 59 visits for older physicians. Among young physicians, EHR use is associated with a decline in patient volume, while EHR use among older physicians is associated with an increase in volume. However, the countervailing effects between younger and older physicians cancel each other out, so that on average, basic EHR use had no statistically significant impact on overall physician visit volume per week at all levels of the volume distribution.

**Table 3:** Quantile regression estimates of the impact of EHR on the physician’s number of patients per week (*N* = 1,470)

Variables	At mean volume	20 visits	64 visits	120 visits
Basic EHR	-0.027 (0.055)	-0.040 (0.160)	-0.072 (0.052)	-0.053 (0.068)
Young physician	0.072 (0.066)	0.303*** (0.109)	0.038 (0.066)	-0.125* (0.074)
Older physician	-0.206*** (0.063)	-0.212 (0.214)	-0.208** (0.088)	-0.158** (0.067)
Basic EHR * Young physician	-0.185 (0.135)	-0.212 (0.309)	-0.094 (0.132)	-0.136 (0.140)
Basic EHR * Older physician	0.403** (0.163)	0.363 (0.452)	0.331** (0.154)	0.390* (0.218)
*N* = 1,470				

Note: Physician-level data. Standard errors are in parentheses. Coefficients on other covariates are available upon request. Number of patients (volume) is logged

***p value < .01

**p value < .05

*p value < .1

## Discussion

In this study, we found that pre-HITECH adoption of EHR among primary care physicians from 2006–2009 was associated with 1.5 extra hours spent on patient face time per week for each physician, with no change in the overall number of visits per week. Since we are using nationally representative data, we estimate that this was 34,000 extra hours of face time per week in the U.S. in 2009 due to EHR. This extra time is likely productive. For example, using the same data, Furukawa has shown that EHR has improved the productivity of an office visit, increasing the number of diagnostic/screening services provided per 20-min period [18]. Another study has found that EHR use increased productivity, measured by the volume and intensity of services per physician workday [24].

While this extra face time may be beneficial to the current patients, EHR does not seem to free up enough time in the week for primary care physicians to see additional patients. This result indicates that EHR adoption is unlikely to help ease the shortage of primary care physicians, especially during the upcoming large insurance expansion under the Patient Protection and Affordable Care Act (ACA). However, this study may provide some insight into how EHR may be adjusted to improve primary care workloads. For one subpopulation of physicians, EHR use did seem to free up enough time for the physicians to take on additional patients. Surprisingly, this efficient subpopulation was the older physicians (age > 61). They expanded visits per week by about 30-40 % whenever they used EHR. This was not simply an artifact of larger practices choosing to adopt EHR, or semi-retired physicians choosing not to adopt EHR. For example, we found this expansion effect among all older physicians, those with only 20 patients a week to those with 120 patients a week.

Another indication that this expansion effect is an efficiency effect of EHR is that older physicians had a dramatically different time management profile than younger physicians who used EHR. At the mean 19 min visit, older and younger physicians were alike, spending an extra 1.5 min of face time per visit due to EHR. However, their time management profiles changed for visits below and above the 19 min mean visit. For 15 min visits, older physicians spent 1.4 min less face time per visit than younger physicians due to EHR, but for 30 min visits, they spent 1.8 min more face time than younger physicians due to EHR. Thus, older physicians appear to be adjusting how they integrate EHR into clinical practice depending on the complexity of the patient visit. In contrast, young physicians always spend 7-9 % extra face time for every type of visit. This might possibly indicate young physicians are spending extra face time on routine EHR elements, such as keyboard entry and check list management. Older physicians might be engaging more complex patients with targeted patient education via the EHR functions. Indeed, these age-based results have been corroborated by a recent article on costs in Massachusetts. Mehrotra et al. (2012) found that physicians with fewer than 10 years of experience had 13.2 % higher overall costs than physicians with 40 or more years of experience. This was not due to malpractice claims or disciplinary actions, board certification status, or the size of the physician group, but appears to be due to older physicians having a more efficient practice style [25]. Thus, we should not be surprised to find that such an efficiency effect also holds with time and workload management among older physicians when they use a new technology such as EHR. Future research should examine how exactly older physicians are integrating the EHR into primary care to improve efficiency.

If younger physicians adopted the practice pattern that we find among older physicians with EHR, we may see some large workforce productivity gains due to EHR. For example, as a very conservative estimate, if young physicians simply increased their number of visits per week from the raw data mean of 62 visits under EHR to that of the older physicians, 74 visits per week under EHR, we would have 37,600 additional patient visits per week in the U.S. Dividing this by 74 visits per physician per week, we conclude that this would be the equivalent of introducing 508 new primary care physicians into the U.S. Thus, the workload productivity of older physicians compared to younger physicians under EHR is equivalent to 508 additional physicians.

Our study has several limitations. First, our findings have limited generalizability and may not be applicable to non-U.S. ambulatory patient visits because the NAMCS only contains information on patient visits to office based physicians in the U.S. Second, the study did not account for physician’s years of experience with EHR which could moderate the association between EHR use and outcomes of interest in the study. Third, we used physician self-reported workloads. It has been shown that physicians often overestimate time spent with the patient [18]. But, since EHR logs the actual time spent, the difference in time between EHR and non-EHR physicians may be larger than what we actually estimate. Next, the use of cross-sectional NAMCS data may not infer a causal association between EHR use and physician’s productivity. It could be that older physicians with larger practices tend to adopt EHRs. However, a quantile regression method allows us to estimate the EHR impacts across the distribution, reflecting the distribution of unobserved characteristics associated with both EHR use and physician’s productivity. Our results from quantile regressions shows that older physicians with any given number of patients, from 20 to 120, always have 30-40 % more patients if they have EHRs, indicative of EHRs possibly driving much of the increased capacity. In addition, our data does not allow assessment of the effects of EHR use on physician’s time spent on non-face time administrative activities. Moreover, our data does not have overall physician hours worked per week (i.e., including administrative time), nor percentages of full time physician employees in clinics, which would allow us to go further in assessing the actual productivity of the physicians. Future research should investigate the causal relation between EHR use and physician’s productivity in administrative work and direct patient care using panel data analyses.

## Conclusion

EHR can enhance productivity/efficiency in primary care physician workloads. Furthermore, the enhancement due to EHR adoption varies across physician ages. Against conventional wisdom, older physicians have higher workload productivity under EHR use, whereas younger physicians, less experienced, but more capable in information technology, have lower workload productivity. Integrating EHR into primary care efficiently depends less on physician information technology capabilities, but more on their experience in clinical practice. There may be substantial efficiency gains to be realized under EHR with better targeted training to younger physicians.

## Availability of data and materials

The datasets supporting the conclusions of this article are available at “http://www.cdc.gov/nchs/ahcd/ahcd_questionnaires.htm” except for the restricted NAMCS data, which is available on request to the Research Data Center, the National Center for Health Statistics, US center for Disease Control and Prevention.

## Appendix 1

**Table 4:** Descriptive Statistics

*Variables*	All visits	Visits with basic EHR	Visits with no basic EHR
Dependent/key independent variables			
Patient face time in minutes (SD)	19.27 (11.37)	20.44 (12.21)	18.97 (11.12)
Number of physician’s patient visits per week^a^ (SD)	69.25 (44.50)	67.50 (35.65)	69.68 (46.45)
Basic EHR use	0.207	1	0
Physician age (SD)	48.97 (10.39)	47.30 (9.61)	49.40 (10.54)
Patient’s characteristics			
Patient age (SD)	47.49 (21.80)	47.76 (22.13)	47.42 (21.71)
Female	0.594	0.596	0.594
Race/ethnicity:			
White, Non-Hispanic	0.644	0.714	0.626
Black, Non-Hispanic	0.135	0.104	0.144
Hispanic	0.093	0.085	0.095
Other race, Non-Hispanic	0.128	0.097	0.136
Patient’s health status			
Major reason for visit:			
New problem	0.409	0.421	0.405
Chronic problem	0.388	0.375	0.392
Pre/post surgery or preventive care	0.203	0.205	0.203
Related to injury/poisoning/adverse effect	0.294	0.211	0.316
Bad disposition (Refer to ED/admit to hospital)	0.007	0.005	0.008
Wound care	0.023	0.022	0.023
Number of new medications (SD)	0.77 (1.06)	0.85 (1.12)	0.74 (1.04)
Number of chronic conditions (SD)	1.38 (1.45)	1.42 (1.48)	1.36 (1.45)
Visit’s characteristics			
Visit to own primary care physician	0.810	0.773	0.820
Referred visit	0.024	0.029	0.023
Primary source of payment:			
Private insurance	0.433	0.516	0.412
Medicare	0.221	0.220	0.221
Medicaid	0.155	0.103	0.168
Other	0.192	0.161	0.199
New patient	0.099	0.107	0.096
Number of visits in last 12 months:			
No visit in last 12 months	0.113	0.133	0.107
1 visit in last 12 months	0.125	0.144	0.120
2 visit in last 12 months	0.178	0.158	0.183
3 or more visit in last 12 months	0.585	0.565	0.590
Weekend visits	0.017	0.022	0.016
Seen also by non-MD clinician	0.469	0.466	0.469
Physician characteristics			
Ownership:			
Physician or physician group	0.565	0.493	0.584
HMO	0.020	0.069	0.008
Community health center	0.275	0.184	0.299
Other type of ownership	0.139	0.254	0.110
Solo practice	0.263	0.140	0.295
Type of office setting:			
Private practice	0.654	0.683	0.646
Free standing	0.038	0.060	0.032
Community health center	0.285	0.198	0.307
HMO	0.016	0.053	0.006
Other type of office setting	0.008	0.007	0.009
Physician’s incentive to change patient face time			
Over 50 % of revenue from capitation	0.039	0.059	0.034
Over 50 % of revenue from managed care contract	0.286	0.389	0.259
Over 50 % of revenue from case rate	0.018	0.013	0.020
Other covariates			
Metropolitan Statistical Area (MSA)	0.850	0.885	0.841
Geographic region:			
Northeast	0.177	0.159	0.182
Midwest	0.262	0.236	0.269
South	0.325	0.304	0.330
West	0.236	0.302	0.219
Survey year:			
2006	0.239	0.129	0.268
2007	0.247	0.223	0.253
2008	0.247	0.277	0.239
2009	0.267	0.371	0.239
N (Patient visits)	39,229	8105	31,124
N (Physicians)^a^	1470	293	1177

Note: Standard deviations are in parentheses

^a^Number of physician’s patient visits per week is in the physician level whereas all other variables are in the visit level

## Appendix 2

**Table 5:** Quantile regression estimates of the impact of EHR on patient face time per visit (All covariates)

Variables	At mean visit	10 minute visit	15 minute visit	20 minute Visit	25 minute Visit	30 minute visit
Key independent variables						
Basic EHR	0.062*** (0.007)	0.053*** (0.012)	0.054*** (0.009)	0.104*** (0.012)	0.112*** (0.011)	0.117*** (0.011)
Young physician	-0.009 (0.008)	0.009 (0.010)	0.010 (0.009)	-0.006 (0.009)	-0.032*** (0.010)	-0.033*** (0.010)
Older physician	0.007 (0.008)	-0.014 (0.012)	-0.001 (0.012)	0.049*** (0.012)	0.026** (0.013)	0.030* (0.018)
Basic EHR * Young physician	0.005 (0.016)	0.016 (0.025)	0.009 (0.021)	-0.027* (0.015)	-0.030 (0.023)	-0.034 (0.024)
Basic EHR * Older physician	0.006 (0.020)	-0.053** (0.021)	-0.073*** (0.026)	0.006 (0.031)	0.020 (0.051)	0.025 (0.048)
Patients’ characteristics						
Age group: (Ref <18)						
18-34	0.054*** (0.009)	0.016 (0.013)	0.045*** (0.015)	0.021** (0.008)	0.075*** (0.017)	0.070*** (0.015)
35-49	0.069*** (0.009)	0.038*** (0.012)	0.069*** (0.014)	0.023*** (0.006)	0.088*** (0.013)	0.079*** (0.014)
50-64	0.082*** (0.009)	0.050*** (0.012)	0.085*** (0.015)	0.044*** (0.009)	0.097*** (0.011)	0.091*** (0.011)
≥ 65	0.072*** (0.011)	0.023* (0.014)	0.066*** (0.017)	0.031*** (0.010)	0.085*** (0.013)	0.080*** (0.017)
Female	0.006 (0.005)	0.007 (0.006)	0.006 (0.006)	0.005 (0.005)	0.017** (0.007)	0.016* (0.009)
Race/ethnicity (Ref: White, non-Hispanic)						
Black, non-Hispanic	-0.039*** (0.007)	-0.012 (0.011)	-0.018 (0.015)	-0.038*** (0.008)	-0.040** (0.016)	-0.044** (0.018)
Other race, non-Hispanic	-0.017** (0.008)	0.006 (0.010)	0.002 (0.008)	-0.019* (0.011)	-0.008 (0.016)	-0.012 (0.017)
Hispanic	-0.001 (0.007)	0.026*** (0.008)	0.019*** (0.006)	-0.010 (0.009)	-0.012 (0.010)	-0.025** (0.012)
Patient’s health status						
Major reason for visit (Ref: New problem)						
Chronic problem	0.052*** (0.005)	0.050*** (0.009)	0.065*** (0.013)	0.049*** (0.007)	0.070*** (0.008)	0.098*** (0.008)
Pre-/post-surgery or preventive care	0.159*** (0.006)	0.092*** (0.012)	0.102*** (0.014)	0.201*** (0.009)	0.295*** (0.009)	0.273*** (0.008)
Related to injury/poisoning/ adverse effect	-0.033*** (0.008)	-0.021** (0.009)	-0.022* (0.012)	-0.030*** (0.006)	-0.040*** (0.015)	-0.043*** (0.015)
Bad disposition (Refer to ED/admit to hospital)	0.151*** (0.026)	0.063 (0.041)	0.063* (0.036)	0.218*** (0.047)	0.287*** (0.033)	0.262*** (0.028)
Wound care	0.139*** (0.015)	0.058** (0.025)	0.058** (0.025)	0.111*** (0.029)	0.196*** (0.032)	0.206*** (0.040)
4+ new medications	0.062*** (0.014)	0.062*** (0.012)	0.052*** (0.013)	0.067*** (0.018)	0.081*** (0.023)	0.064*** (0.023)
1 chronic conditions	0.027*** (0.006)	0.013 (0.009)	0.022* (0.012)	0.021*** (0.005)	0.036*** (0.008)	0.035*** (0.010)
2 chronic conditions	0.050*** (0.007)	0.059*** (0.013)	0.057*** (0.012)	0.050*** (0.014)	0.049*** (0.012)	0.061*** (0.014)
3+ chronic conditions	0.074*** (0.008)	0.072*** (0.013)	0.068*** (0.013)	0.117*** (0.015)	0.095*** (0.015)	0.094*** (0.015)
Visit’s characteristics						
Visit to own primary care physician	0.017** (0.007)	0.030*** (0.008)	0.034*** (0.006)	0.024*** (0.009)	0.003 (0.014)	-0.020 (0.017)
Referred visit	0.087*** (0.016)	-0.005 (0.019)	-0.001 (0.016)	0.114*** (0.027)	0.121*** (0.024)	0.091*** (0.021)
Primary source of payment (Ref: Private insurance)						
Medicare	-0.001 (0.008)	0.023*** (0.008)	0.022** (0.010)	-0.005 (0.011)	0.002 (0.014)	0.006 (0.017)
Medicaid	-0.005 (0.008)	-0.021* (0.011)	-0.007 (0.014)	0.000 (0.007)	0.002 (0.012)	0.015 (0.013)
Other	0.009 (0.007)	0.015* (0.009)	0.017** (0.008)	0.002 (0.006)	0.017* (0.009)	0.029** (0.013)
New patient	0.113*** (0.011)	0.043*** (0.015)	0.067*** (0.014)	0.160*** (0.023)	0.165*** (0.019)	0.155*** (0.020)
Number of visits in last 12 months (Ref: No visit)						
1 visit	-0.010 (0.012)	-0.011 (0.015)	-0.006 (0.016)	-0.021 (0.015)	-0.002 (0.015)	-0.003 (0.023)
2 visits	-0.031*** (0.012)	-0.027** (0.013)	-0.018 (0.013)	-0.027* (0.015)	-0.024 (0.016)	-0.031 (0.022)
3+ visits	-0.031*** (0.010)	-0.021 (0.014)	-0.014 (0.012)	-0.034** (0.014)	-0.040*** (0.013)	-0.044** (0.019)
Weekend visit	-0.053*** (0.017)	-0.052** (0.026)	-0.082** (0.035)	-0.019 (0.015)	-0.068*** (0.022)	-0.058 (0.035)
Seen also by non-MD clinician	-0.003 (0.005)	-0.006 (0.005)	-0.003 (0.006)	-0.011 (0.007)	-0.006 (0.008)	0.003 (0.010)
Physician characteristics						
Ownership (Ref: physician or physician group)						
HMO	0.070*** (0.020)	0.094*** (0.032)	0.057*** (0.018)	0.102*** (0.021)	0.024 (0.022)	0.036 (0.037)
Community health center	-0.075*** (0.021)	-0.040*** (0.012)	-0.057*** (0.016)	-0.039** (0.019)	-0.060** (0.024)	-0.086*** (0.032)
Other type of ownership	-0.033*** (0.007)	-0.043*** (0.012)	-0.049*** (0.016)	-0.016* (0.009)	-0.034*** (0.009)	-0.042*** (0.011)
Solo practice	0.058*** (0.006)	0.022*** (0.006)	0.031*** (0.006)	0.065*** (0.007)	0.077*** (0.008)	0.074*** (0.010)
Type of office setting (Ref: Private practice)						
Free standing	0.001 (0.012)	0.045** (0.019)	0.059*** (0.010)	-0.009 (0.015)	-0.051*** (0.013)	-0.092*** (0.018)
Community health center	0.090*** (0.020)	0.080*** (0.016)	0.094*** (0.017)	0.077*** (0.023)	0.067*** (0.024)	0.082** (0.032)
HMO	-0.087*** (0.022)	-0.170*** (0.052)	-0.098*** (0.034)	-0.049** (0.020)	-0.084*** (0.020)	-0.103*** (0.029)
Other type of office setting	0.133*** (0.026)	0.160*** (0.031)	0.140*** (0.021)	0.132*** (0.045)	0.159*** (0.059)	0.134*** (0.034)
Physician’s incentive to change patient face time						
Over 50 % of revenue from capitation	-0.057*** (0.012)	-0.041*** (0.014)	-0.038** (0.018)	-0.047*** (0.009)	-0.099*** (0.015)	-0.101*** (0.022)
Over 50 % of revenue from managed care contract	0.009 (0.005)	0.028*** (0.010)	0.048*** (0.008)	-0.002 (0.006)	-0.008 (0.007)	-0.016** (0.008)
Over 50 % of revenue from case rate	0.040** (0.017)	0.005 (0.024)	-0.019 (0.026)	0.078*** (0.029)	0.081** (0.041)	0.105*** (0.022)
Other covariates						
Metropolitan Statistical Area (MSA)	0.018*** (0.007)	0.034*** (0.009)	0.022* (0.013)	0.007 (0.007)	-0.012 (0.011)	-0.022** (0.011)
Geographic Region (Ref: Northeast)						
Midwest	0.001 (0.007)	-0.056*** (0.018)	-0.092*** (0.021)	0.002 (0.010)	0.019* (0.010)	0.020* (0.011)
South	0.005 (0.007)	-0.042** (0.019)	-0.024 (0.016)	0.009 (0.011)	0.036*** (0.011)	0.040*** (0.011)
West	0.064*** (0.007)	0.165*** (0.022)	0.069*** (0.011)	0.014 (0.010)	0.064*** (0.012)	0.080*** (0.013)
*N* = 37,962						

Note: Visit-level data. Standard errors are in parentheses. Survey year dummies are not shown. Minutes are logged

****p* value < .01

***p* value < .05

**p* value < .1

## Footnotes

1 For example, at 10 min visits for young physicians, the full impact of EHR from Table 2 is the sum of the coefficients: EHR + EHR*young = .053 + .016 = .069. Not shown, the standard error for this sum is estimated to be .017, so the full effect of 0.069 has a p-value < 0.001. This 0.069 is then log retransformed, giving 0.071 as the full effect of EHR on 10 min visit time for young physicians. This 0.071 effect is the point plotted in Fig. 1 for young doctors at 10 min visits. Similar computations underlie all the final full effects of EHR plotted in Exhibits 2 and 3.

2 For example, for the young physicians, the full effect of EHR in Table 2, col 1, is the sum of the coefficients: EHR + EHR*young = .067 (SE = .015). Log retransforming 0.067 gives a 7 % effect.

## References

[1] Davis K, Schoenbaum SC, Audet AM (2005). A 2020 vision of patient‐centered primary care. J Gen Intern Med.

[2] Rittenhouse DR, Shortell SM (2009). The patient-centered medical home: will it stand the test of health reform?. JAMA.

[3] Goldsmith J (2011). Accountable care organizations: the case for flexible partnerships between health plans and providers. Health Aff.

[4] Petterson SM, Liaw WR, Phillips RL, Rabin DL, Meyers DS, Bazemore AW (2012). Projecting US primary care physician workforce needs: 2010-2025. Ann Fam Med.

[5] U.S. Senate Subcommittee on Primary Health and Aging. 2013. Primary Care Access. http://www.sanders.senate.gov/imo/media/doc/PrimaryCareAccessReport.pdf. Accessed 4 May 2016.

[6] Yarnall KS, Østbye T, Krause KM, Pollak KI, Gradison M, Michener JL (2009). Family physicians as team leaders: “time” to share the care. Prev Chronic Dis.

[7] Center for Studying Health System Change. 2008. Physician Survey. http://hscdataonline.s-3.com/psurvey.asp. Accessed 4 May 2016.

[8] AHRQ (Agency for healthcare Research and Quality) (2012). The 2012 CAHPS database: the CAHPS clinician & group survey: Adult 12-Month 6-pt Scale Results.

[9] Burdi MD, Baker LC (1999). Physicians’ perceptions of autonomy and satisfaction in California. Health Affair.

[10] Lin CT, Albertson GA, Schilling LM (2001). Is patients’ perception of time spent with the physician a determinant of ambulatory patient satisfaction?. Arch Intern Med.

[11] Gross DA, Zyzanski SJ, Borawski EA, Cebul RD, Stange KC (1998). Patient satisfaction with time spent with their physician. J Fam Pract.

[12] Chaudhry B, Wang J, Wu S (2006). Systematic review: impact of health information technology on quality, efficiency, and costs of medical care. Ann Intern Med.

[13] Orszag PR. Evidence on the costs and benefits of health information technology. Testimony before the Subcommittee on Health Committee on Ways and Means U.S. House of Representative. Congressional Budget Office: Washington, DC. July 24, 2008. http://www.cbo.gov/sites/default/files/cbofiles/ftpdocs/95xx/doc9572/07-24-healthit.pdf. Accessed 4 May 2016.

[14] Poissant L, Pereira J, Tamblyn R, Kawasumi Y (2005). The impact of electronic health records on time efficiency of physicians and nurses: a systematic review. J Am Med Inform Assoc.

[15] Centers for Medicare and Medicaid Services. Active Registrations: EHR Incentive Program. http://www.cms.gov/Regulations-and-Guidance/Legislation/EHRIncentivePrograms/Downloads/June_Medicare_EHRIncentivePayments.pdf. Accessed 4 May 2016.

[16] Xierali IM, Hsiao CJ, Puffer JC (2013). The rise of electronic health record adoption among family physicians. Ann Fam Med.

[17] Pizziferri L, Kittlera AF, Volka LA (2005). Primary care physician time utilization before and after implementation of an electronic health record: a time-motion study. J Biomed Inform.

[18] Furukawa MF (2011). Electronic medical records and efficiency and productivity during office visits. Am J Manag Care.

[19] Gilchrist V, McCord G, Schrop SL (2005). Physician activities during time out of the examination room. Ann Fam Med.

[20] Farber J, Siu A, Bloom P (2007). How much time do physicians spend providing care outside of office visits?. Ann Intern Med.

[21] Hsiao CJ, Cherry DK, Beatty PC (2010). National Ambulatory Medical Care Survey: 2007 summary. Natl Health Stat Rep.

[22] DesRoches CM, Campbell EG, Rao SR (2008). Electronic health records in ambulatory care-a national survey of physicians. N Engl J Med.

[23] Greene W (2012). Econometric analysis.

[24] Adler-Milstein J, Huckman RS (2013). The impact of electronic health record use on physician productivity. Am J Manag Care.

[25] Mehrotra A, Reid RO, Adams JL (2012). Physicians with the least experience have higher cost profiles than do physicians with the most experience. Health Aff.

